# Suspected congenital hyperinsulinism in a Shiba Inu dog

**DOI:** 10.1111/jvim.15834

**Published:** 2020-06-27

**Authors:** Simon Cook, Myles McKenna, Barbara Glanemann, Ranbir Sandhu, Chris Scudder

**Affiliations:** ^1^ Department of Clinical Science and Services Royal Veterinary College London United Kingdom; ^2^ London United Kingdom; ^3^ Department of Comparative Biomedical Sciences Royal Veterinary College London United Kingdom; ^4^ Southfields Veterinary Specialists Laindon, Essex United Kingdom

**Keywords:** hypoglycemia, inherited, insulinoma, nesidioblastosis, pancreatic hyperplasia

## Abstract

A 3‐month‐old male intact Shiba Inu dog was evaluated for a seizure disorder initially deemed idiopathic in origin. Seizure frequency remained unchanged despite therapeutic serum phenobarbital concentration and use of levetiracetam. The dog was documented to be markedly hypoglycemic during a seizure episode on reevaluation at 6 months of age. Serum insulin concentrations during hypoglycemia were 41 U/μL (reference range, 10‐29 U/μL). The dog was transitioned to 4 times per day feeding, diazoxide was started at 3.5 mg/kg PO q8h, and antiepileptic drugs were discontinued. No clinically relevant abnormalities were identified on bicavitary arterial and venous phase contrast computed tomographic imaging. The dog remained seizure‐free and clinically normal at 3 years of age while receiving 5.5 mg/kg diazoxide PO q12h and twice daily feeding. Seizures later occurred approximately twice per year and after exertion, with or without vomiting of a diazoxide dose. Blood glucose curves and interstitial glucose monitoring were used to titrate diazoxide dose and dosing interval. Congenital hyperinsulinism is well recognized in people but has not been reported in veterinary medicine.

**Abbreviations**
CHI: congenital hyperinsulinism
IGF: insulin‐like growth factor

## 1 CASE DESCRIPTION

A 3‐month‐old intact male Shiba Inu was presented for evaluation of seizure episodes. Seizures involved focal symmetrical facial and auricular twitching and tonic episodes affecting all 4 limbs with opisthotonos, urination and defecation, and progression to generalized tonic‐clonic seizures. The dog often was normal by the time of examination but was reported to be lethargic after seizures for several minutes. These events occurred once weekly and lasted from 1 to 30 minutes. Episodes tended to occur in the early hours of the morning. General physical examination was unremarkable and the dog appeared to be growing appropriately. Neurological examination was unremarkable except for occasional aggressive behavior. The history of seizures was considered consistent with a forebrain neuroanatomical localization.

Investigations during 3 visits over the course of 10 days documented both normoglycemia (77 mg/dL [Vetscan VS2, Abaxis; reference range, 59‐110 mg/dL; visit 2] and 81 mg/dL [Alphatrack 2, Zoetis; visit 2]) and borderline or actual hypoglycemia with recordings of 68 mg/dL (Alphatrack 2, visit 1), 65 mg/dL (Radiometer ABL 800 Flex; reference range, 76‐119 mg/dL; visit 3), and 49 mg/dL (VetScan VS2; reference range, 59‐110 mg/dL; visit 1). Plasma ammonia concentration was 15 μM/L (reference range, 0‐99 μM/L). Blood gas analysis, hematology, and serum biochemistry results were otherwise normal. Serum cholesterol concentration was 5.42 mM/L (reference range, 3.2‐6.2 mM/L) and although serum triglyceride concentration was not measured, lipemic serum was not reported. Bile acid stimulation testing was normal (preprandial, 1.9 μM/L; reference range, 0‐25 μM/L; postprandial, 5.1 μM/L; reference range, 0‐40 μM/L) and serology testing for *Toxoplasma gondii* and *Neospora caninum* was negative. Urine specific gravity was 1.029, no glucosuria was detected, and sediment examination was normal. Urine culture was not performed.

**FIGURE 1:**
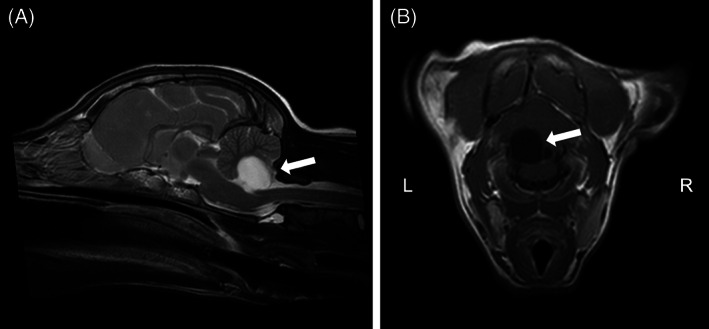
T2‐weighted sagittal (A) and T1‐weighted, transverse (at the level of the caudal medulla oblongata), postcontrast (B) magnetic resonance images. A focal, cystic lesion (arrow) creates mild compression of the left cerebellar hemisphere and vermis. The lesion is T2W hyperintense compared to normal gray matter (isointense to cerebrospinal fluid), and T1W hypointense with no contrast enhancement

Borderline hypoglycemia was thought to represent juvenile fasting hypoglycemia. The dog was anesthetized and high‐field magnetic resonance imaging of the head performed (1.5T Intera, Philips Medical Systems, Eindhoven, the Netherlands). Sagittal and transverse plane T2‐weighted turbo‐spin echo (T2W TSE), fluid attenuated inversion recovery (FLAIR), transverse plane T2* gradient echo, and sagittal and transverse T1‐weighted (T1W TSE) images were acquired before and after IV injection of gadolinium contrast (0.1 mM/kg gadobutrol; Bayer plc, Strawberry Hill, UK). Imaging identified a focal, well‐demarcated, cystic caudal fossa lesion ventral to the cerebellum on the left side of the fourth ventricle. The lesion was T2W hyperintense compared to normal gray matter (isointense to cerebrospinal fluid), T1W hypointense compared to normal gray matter and FLAIR attenuating with no contrast enhancement. The lesion created a mass effect with compression of the left cerebellar hemisphere and vermis, and a mild dorsal compression of the left side of the medulla oblongata. No other abnormalities were detected. The lesion was considered unrelated to the presumed forebrain localization and an epithelial or ependymal cyst was considered most likely (Figure 1). No clinical signs attributable solely to a compressive lesion at this site had been observed. The dog was treated with phenobarbital at 3 mg/kg PO q12h and subsequently levetiracetam at 20 mg/kg PO q8h, which later was increased to 30 mg/kg PO q8h because of a cluster of seizures 8 weeks later. Serum phenobarbital concentration was monitored and the dosage of phenobarbital titrated in accordance with physiological weight gain, achieving a therapeutic concentration (73.3 μM/L; reference range, 65‐194 μM/L) at a dosage of 3.5 mg/kg PO q12h, without any apparent improvement in seizure control.

At 6 months of age, the dog was presented again during a facial twitching episode. These events had still been occurring intermittently (approximately every 2 weeks) since 3 months of age despite antiepileptic treatment and progressed to truncal and limb twitching, also with occasional generalized tonic episodes. Episodes occurred at any time of day but often overnight, and the dog was otherwise normal. At this time, the dog was documented to be markedly hypoglycemic (29‐45 mg/dL; reference range, 76‐119 mg/dL) during 2 days of hospitalization. Clinical signs resolved immediately after IV administration of 0.25 g/kg dextrose. The frequency and severity of clinical signs both were decreased with frequent feeding during hospitalization.

Additional investigations for the cause of hypoglycemia included an ACTH stimulation test (pre‐ACTH plasma concentration of cortisol 0.7 μg/dL and post‐ACTH plasma concentration of cortisol 15 μg/dL), measurement of serum total T4 concentration (11.3 nM/L; reference range, 10‐55 nM/L) and serum thyroid stimulating hormone concentration (0.06 ng/mL; reference range, 0.01‐0.6 ng/mL), serum insulin concentration (41 U/mL; reference range 11.6‐29 U/mL) with concurrent blood glucose concentration of 54 mg/dL (Alphatrack 2) and 58 mg/dL (fluoride oxalate sample; external laboratory reference range, 63‐108 mg/dL) and serum IGF‐1 concentration of 472 μg/L (reference ranges: dwarfism, <50 μg/L, acromegaly, >1000 μg/L). Urine culture yielded no growth. Serum fructosamine concentration was not measured.

The dog was treated with diazoxide (Eudamine, RPH Pharmaceuticals, Jordbro, Sweden) at a dosage of 3.5 mg/kg PO q8h. Feeding also was divided into 3 to 4 meals per day, using Royal Canin diabetic diet (Royal Canin, Gard, France) to minimize postprandial insulin surges. No clinically relevant abnormalities were found on arterial and venous phase computed tomography imaging of the abdomen and thorax (16‐slice CT scanner [General Electric Medical Systems, Lightspeed, Chicago, Illinois] and power injector [Empower CTA, Acist Medical Systems, Eden Prairie, Minnesota]). Levetiracetam treatment was discontinued and phenobarbital was tapered over 9 weeks and then discontinued. The dog was neutered at 8 months of age because testosterone may increase insulin sensitivity,^1^, ^2^ but no clinical benefit was appreciated after the procedure.

The dog's clinical signs initially were controlled (mild facial twitching episodes once every few weeks) when receiving 4.5 mg/kg diazoxide PO q12h and twice daily feeding. An orally administered glucose supplement (Glucostop, British Biocell International, UK) was utilized when clinical signs of hypoglycemia were observed. Diazoxide dose adjustments were made as the dog gained weight with maturity, and in response to clinical signs. At 18 months of age, imaging was repeated, and exploratory surgery with pancreatic biopsy was discussed but not pursued. No abnormalities were detected on repeated arterial and venous phase contrast CT imaging (320‐slice CT scanner [Canon Medical Systems, Aquilion ONE Genesis Edition, Tochigi, Japan] and power injector [Bayer plc, Medrad Stellant, Leverkusen, Germany]. FreeStyle Libre (Abbot Laboratories Limited, UK) interstitial glucose recording at this time documented average daily blood glucose concentrations of 74 to 110 mg/dL, during a period when the dog had seizure episodes approximately biannually (focal or generalized), on a diazoxide dosage of 5.5 mg/kg PO q12h and consequently this dose was maintained. The dog's growth, conditioning, and muscle mass remained normal.

A transient inability to obtain diazoxide resulted in transition to prednisolone (0.5 mg/kg PO q12h) for a period of 3 weeks. During this time, a repeat 24‐hour blood glucose curve documented blood glucose concentrations of 50 to 88 mg/dL in addition to subdued demeanor attributed to hypoglycemia. The dog also was poorly tolerant of the glucocorticoid treatment, with polyphagia, polyuria and polydipsia, and a 1 kg weight loss. Diazoxide was reinstituted and the dog remains clinically normal on 5.5 mg/kg PO q12h of diazoxide, at 3 years of age. Rare seizures are observed approximately twice per year (focal or generalized) after exertion, and with or without vomiting of a diazoxide dose.

## 2 DISCUSSION

We describe the presentation and management of a juvenile dog with hyperinsulinemic hypoglycemic syndrome. Although neither genotyping nor pancreatic histopathological evaluation was available, the signalment, investigations performed and stability on medical treatment over 2 years are inconsistent with insulinoma, and parallel a diazoxide‐responsive form of the syndrome of congenital hyperinsulinism (CHI) in people.

A clinical diagnosis of insulinoma requires documentation of hyperinsulinemia with hypoglycemia, imaging of the pancreas, and often surgical exploration if abdominal imaging does not identify a focal pancreatic lesion.^3^ Considering signalment and history, the index of suspicion usually is high enough for a neoplastic process that surgical exploration, intraoperative imaging and partial pancreatectomy are warranted, with histopathology used as a confirmatory test. Computed tomography has been reported to have a sensitivity of 71% for identifying pancreatic insulinomas^4^ and dual phase contrast CT now is recommended^5^ for increased sensitivity. Repeated dual phase contrast CT identified no such abnormalities in this dog, and thus hyperinsulinism was presumed to result from a diffuse pancreatopathy. Furthermore, the previously reported age range of dogs with insulinoma is 4 to 14 years^6^, ^7^ and in 50% of cases the disease is already metastatic at the time of presentation.^8^ Although survival times of 2 to 4 years have been reported after combined surgical and medical management of insulinoma,,^8^ medical treatment alone is associated with significantly shorter median survival times when compared to surgical and follow‐up medical treatment, with the longest reported survival of a patient receiving medical management only being 549 days.^6^, ^9^ A diagnosis of insulinoma was excluded based on absence of nodular pancreatic lesions on 2 CT examinations, signalment and long‐term stability without relapse.

In people, hyperinsulinism in infancy can be transient or persistent. Transient causes of hyperinsulinism include being the infant of a diabetic mother, perinatal stress or asphyxia, erythroblastosis fetalis, sepsis, and umbilical artery catheter placement. Hyperinsulinism in these situations tends to resolve within days to weeks.^10^ A number of overlapping and outdated terms have been used to describe juvenile, persistent, hyperinsulinemic hypoglycemia, such as nesidioblastosis (also used to describe an adult‐onset phenotype), persistent hyperinsulinemic hypoglycemia in infancy and idiopathic hypoglycemia of infancy. Congenital hyperinsulinism is now the preferred term, incorporating a growing number of characterized mutations, most often of genes encoding 2 components of the pancreatic β‐cell ATP‐sensitive plasma membrane potassium (KATP) channel: the sulfonylurea receptor 1 (SUR1) and Kir6.2 protein.^11^ Dysfunctional or absent KATP activity results in persistent membrane depolarization and insulin release, unrelated to plasma glucose concentrations. The type of mutation affects the number or activity of the KATP channels, and hence some mutations may result in diazoxide‐responsive hypoglycemia; the extent of residual channel activity dictating diazoxide responsiveness. Some of the more rare mutations alter the intracellular ATP concentration that induces KATP closure, membrane depolarization, and insulin release independently of blood glucose concentration.

Congenital hyperinsulinism is the most common cause of persistent hyperinsulinemic hypoglycemia in infants^12^ and can occur in both focal and diffuse forms. The focal form lends itself to partial pancreatectomy, but the focal nature is challenging to confirm, requiring genotyping, fluorine‐18 L‐3,4‐dihydroxyphenylalanine positron emission tomography,^13^ intraoperative ultrasonography,^14^ intraoperative frozen section histopathology,^15^ or some combination of these for confirmation. The diffuse form may require subtotal pancreatectomy as part of management, but it is infrequently curative.^15^, ^16^

Diazoxide responsiveness is an important feature used in people to allow characterization of CHI. Among diazoxide‐responsive CHI patients, a genetic mutation is only identified in 27% to 47% of patients. Among those with mutations identified, the most common are KATP channel mutations, GLUD1 mutations (causing glutamate dehydrogenase dysfunction and hyperinsulinism with hyperammonemia) and GCK (glucokinase) mutations, with the remainder being rare mutations in genes such as HNF4A, HADH, UCP2, and HNF1A.^11^, ^17^ Patients without identifiable mutations may experience spontaneous resolution over time, but the timescale of resolution is unpredictable.^18^ This same study suggested that lack of an identifiable genetic mutation and a response to diazoxide are associated with disease remission.

Despite a positive response to diazoxide treatment, insufficient evidence was available to confirm an etiology in our case. Two cases termed nesidioblastosis have been reported in the veterinary literature (a 6‐year‐old cat and a 6‐year‐old dog).^19^, ^20^ In these cases, adult‐onset hypoglycemia resolved with partial pancreatectomy in a dog, and pancreatic histopathology disclosed diffusely increased islet area in the pancreas without evidence of malignancy and multifocal micronodular hyperplasia of endocrine and exocrine tissue in a cat. Both cases were managed successfully by partial pancreatectomy.

Infectious diseases, such as bartonellosis and babesiosis, have been reported as causes of hyperinsulinemic hypoglycemia in dogs. The dog of our report was not tested for these diseases because of systemic stability and a low index of clinical suspicion.^21^, ^22^ Other differential diagnoses excluded or considered unlikely were growth hormone deficiency (based on normal growth and a normal serum IGF‐1 concentration), glycogen storage diseases (type 1 [von Gierke's disease] or type III [Cori's disease]),^23^ and hyperketotic hypoglycemia. These glycogen storage diseases would tend to manifest with a more severe and progressive clinical picture, including weakness, failure to thrive, exercise intolerance, and hepatopathy.^24^, ^25^, ^26^ Hyperketotic hypoglycemia encompasses several etiologies including an idiopathic form.^27^ Although β‐hydroxybutyrate was not measured, no acidosis suggestive of marked ketonemia was documented and no ketonuria was observed. Hyperammonemia hypoglycemia syndrome, caused by a glutamate dehydrogenase mutation, was considered but deemed unlikely based on normal plasma ammonia concentration during initial clinical investigations. Congenital glucagon deficiency is poorly described but tends to be associated with normal insulin concentrations and normal insulin regulation.^28^, ^29^ In patients with CHI, production of glucagon in response to hypoglycemia appears to be blunted,^30^ but the capacity for primary glucagon deficiency to cause hypoglycemia in the face of intact hypothalamic‐pituitary‐adrenal and sympathetic adrenomedullary axes is debated.^31^, ^32^ Counter‐regulatory hormone release and antagonism of insulin activity likely explain rebound normoglycemia on presentation on multiple occasions in our dog.^33^

Diazoxide is a thiazide drug with vasodilatory (and therefore antihypertensive) properties but without diuretic activity. It inhibits pancreatic insulin secretion by binding to the SUR1 subunit of β‐cell KATP channels and activating (opening) them, limiting intracellular calcium‐mediated insulin release, thereby promoting hyperglycemia.^34^ Recognized adverse effects include inappetence. Long‐term use has not been specifically reported, but the dog described here appears to have been tolerant for 2 years without ongoing clinical signs beyond initial, transient inappetence. Dosing is recommended at 3.3 mg/kg PO q8h up to 20 to 30 mg/kg PO q8h.^35^, ^36^ Consideration also could be given to the use of somatostatin analogues (eg, octreotide) in the dog of this report, should management of clinical signs prove problematic in the future, or during any further supply difficulties.^37^

## 3 SUMMARY

Although a relatively novel and extremely rare disease, CHI (of multiple etiologies) is a differential diagnosis for persistently hypoglycemic juvenile dogs, and even may be considered in normoglycemic patients with a history of seizures as evidenced by our dog and the dog of previous case report.^19^ Medical management with diazoxide has proven successful thus far, but surgical management and even spontaneous disease remission both remain possibilities. The affected dog appears to have a good midrange to long‐term prognosis.

## CONFLICT OF INTEREST DECLARATION

Authors declare no conflict of interest.

## OFF‐LABEL ANTIMICROBIAL DECLARATION

Authors declare no off‐label use of antimicrobials.

## INSTITUTIONAL ANIMAL CARE AND USE COMMITTEE (IACUC) OR OTHER APPROVAL DECLARATION

Authors declare no IACUC or other approval was needed.

## HUMAN ETHICS APPROVAL DECLARATION

Authors declare human ethics approval was not needed for this study.
